# Reconstitution of the Melibiose Permease of *Salmonella enterica* serovar Typhimurium (MelB_St_) into Lipid Nanodiscs

**DOI:** 10.21769/BioProtoc.5045

**Published:** 2024-08-05

**Authors:** Parameswaran Hariharan, Lan Guan

**Affiliations:** Dept of Cell Physiology and Molecular Biophysics, Center for Membrane Protein Research, School of Medicine, Texas Tech University Health Sciences Center, Lubbock, TX, USA

**Keywords:** MelB_St_, Membrane scaffold proteins, Major facilitator superfamily, Membrane proteins, Nanodiscs, Cryo-EM single-particle analysis, Reconstitution with lipids

## Abstract

Membrane proteins play critical roles in cell physiology and pathology. The conventional way to study membrane proteins at protein levels is to use optimal detergents to extract proteins from membranes. Identification of the optimal detergent is tedious , and in some cases, the protein functions are compromised. While this detergent-based approach has produced meaningful results in membrane protein research, a lipid environment should be more suitable to recapture the protein’s native folding and functions. This protocol describes how to prepare amphipathic membrane scaffold-proteins (MSPs)-based nanodiscs of a cation-coupled melibiose symporter of *Salmonella enterica* serovar Typhimurium (MelB_St_), a member of the major facilitator superfamily. MSPs generate nano-assemblies containing membrane proteins surrounded by a patch of native lipids to better preserve their native conformations and functions. This protocol requires purified membrane protein in detergents, purified MSPs in solution, and detergent-destabilized phospholipids. The mixture of all three components at specific ratios is incubated in the presence of Bio-Beads SM-2 resins, which absorb all detergent molecules, allowing the membrane protein to associate with lipids surrounded by the MSPs. By reconstituting the purified membrane proteins back into their native-like lipid environment, these nanodisc-like particles can be directly used in cryo-EM single-particle analysis for structure determination and other biophysical analyses. It is noted that nanodiscs may potentially limit the dynamics of membrane proteins due to suboptimal nanodisc size compared to the native lipid bilayer.

Key features

• This protocol was built based on the method originally developed by Sligar et al. [1] and modified for a specific major facilitator superfamily transporter

• This protocol is robust and reproducible

• Lipid nanodiscs can increase membrane protein stability, and reconstituted transporters in lipid nanodiscs can regain function if their function is compromised using detergents

• The reconstituted lipids nanodisc can be used for cryo-EM single-particle analysis

## Graphical overview

**Figure 1.**
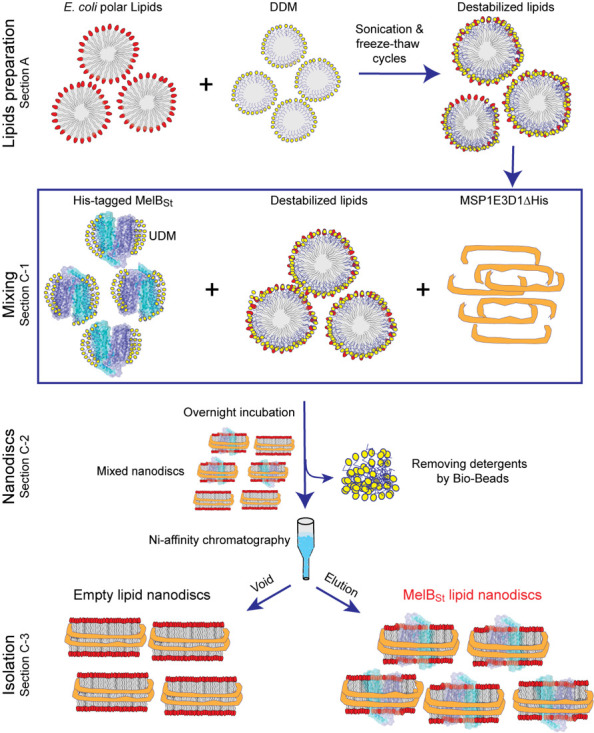
Overview of the protocol to prepare MelB_St_ lipid nanodiscs . Section A illustrates the preparation of detergent-destabilized lipids. Section C-1 shows the steps for mixing the detergent-stabilized lipids with purified MelB_St_ and then with purified membrane scaffold protein MSP1E3D1. Section C-2 illustrates the formation of lipid nanodiscs upon incubation with detergent-adsorbing Bio-Beads SM2. Section C-3 illustrates the Ni^+^-agarose resin affinity chromatography to isolate the reconstituted MelB_St_ nanodiscs. DDM, n-Dodecyl-β-D-maltopyranoside; UDM, n-Undecyl-β-D-maltopyranoside.

## Background

Membrane proteins and phospholipids are two major essential components of cell membranes. Lipid-embedded membrane proteins play critical roles in supplying nutrients and extruding unwanted or toxic substances by providing specific paths that allow polar or charged molecules across the cell membrane at a rate that effectively meets cellular needs [2]. The functions of membrane proteins are carried out in the lipid environment, and their activities are modulated or regulated by lipids [3]. The interactions between membrane proteins and phospholipids, including cholesterol, are dynamic and complicated, and some membrane proteins have binding site(s) to tightly interact with specific lipids [4,5]. The roles of lipids on membrane proteins vary from stabilization and oligomerization to modulating conformational dynamics [6,7]. This is a poorly characterized research area due to technical challenges stemming from intrinsic hydrophobicity. The conventional way to conduct the study of membrane proteins at protein levels is to use optimal detergents to extract proteins from the membranes for their function analyses. Identification of the optimal detergent is tedious and often challenging. Accordingly, varied methods have been developed to overcome this problem [8–10]; one is to reconstitute the protein back to a lipid environment after it has been purified, such as proteoliposomes and lipid nanodiscs [1,11–13]. The membrane scaffold protein (MSP)-based lipid nanodiscs [1] contain a lipid-bilayer core around membrane proteins, which retain and provide a more native-like environment than detergent micelles. In most cases, those protein nanodiscs contain a single copy of membrane protein per particle. The nanodisc-like samples have successfully facilitated cryo-EM single-particle reconstruction and membrane biophysical analyses. Reconstitution into the native-like environments allows some membrane proteins to regain function. For example, a protein that lost its function when solubilized in the detergents can bind ligands again after being reconstituted into proteoliposomes or lipid nanodiscs [5,14–16].

Compared to proteoliposomes, the nanodisc form lacks membrane-sidedness, and the membrane proteins are exposed to bulk solvents at both hydrophilic surfaces. This also means that the nanodisc form cannot be used to test the transport activity of the reconstituted transporters since no boundary exists. It is also noted that nanodiscs may potentially limit the dynamics of membrane proteins if their size is suboptimal.

In this protocol, we describe with great detail the reconstitution of a cation-coupled melibiose symporter of *Salmonella enterica* serovar Typhimurium (MelB_St_) into nanodisc form (Figure 1), which has been validated by cryo-EM single-particle analysis and biophysical analysis [17,18]. MelB_St_ is a member of the major superfamily of transporters with 12 transmembrane helices embedded in the membrane [19]. This family of proteins is dominated by a hydrophobic domain with limited hydrophilic surfaces. Maintaining the protein's stability and function after extraction from native membranes is often challenging. MelB_St_ catalyzes the symport of a galactopyranoside with either H^+^, Li^+^, or Na^+^, and it is a unique model system for studying cation-coupled transport mechanisms [17,20–22]. This nanodisc protocol, developed for studying this protein, is robust and reproducible, and was originally developed by Sligar [1] with a modified lipids preparation procedure as described [23].

## Materials and reagents

**Reagents**

1. *E. coli* polar lipids extract (Avanti, catalog number: 100500P)
2. n-Dodecyl-β-D-maltopyranoside (DDM) powder (Anatrace, catalog number: D310)
3. n-Undecyl-β-D-maltopyranoside (UDM) (Anatrace, catalog number: U300)
4. Bio-Beads SM-2 adsorbents (Bio-Rad, catalog number: 1523920)
5. Triton X-100 (Sigma-Aldrich, catalog number: X100)
6. Sodium chloride (NaCl) (Fisher bioreagents, catalog number: S271)
7. Tris base (RPI, catalog number: T60040)
8. Concentrated HCl (Fisher Chemical, catalog number: A144-212)
9. Imidazole (Acros Organics, catalog number: 301870010)
10. Glycerol (Fisher Bioreagents, catalog number: BP229)
11. Methanol (Fisher Chemical, catalog number: A452SK-4)
12. Ethanol (Fisher bioreagents, catalog number: BP28184)
13. MSP1E3D1∆His at 260 μM (purified by nickel-based affinity chromatography using INDIGO Ni-Agarose resin and processed by TEV protease digestion; alternative source, Cube Biotech, catalog number: 26162 and concentrated by centrifugal concentrator Vivaspin 20, 5,000 MWCO)
14. His-tagged MelB_St_ (purified by cobalt-based affinity chromatography using TALON^®^ Metal Affinity Resin)
15. Liquid nitrogen (Airgas)

**Solutions**

1. 0.001% Triton X100 bath liquid (see Recipes)
2. 7.5% DDM (see Recipes)
3. MelB_St_ dialysis buffer (see Recipes)
4. TBS (see Recipes)
5. TBS + 5 mM imidazole buffer (column binding buffer) (see Recipes)
6. TBS + 25 mM imidazole buffer (column wash buffer) (see Recipes)
7. TBS + 300 mM imidazole buffer (column elution buffer) (see Recipes)
8. 1 M Tris-HCl (see Recipes)
9. 1 M imidazole (see Recipes)

**Recipes**

1. **0.001% (v/v) Triton X100 bath liquid**
  **Table:** 

Reagent	Final concentration	Amount
100% Triton X-100	0.001%	0.05 mL
Milli-Q H_2_O*	n/a	To 5 L
  *Milli-Q H_2_O is prepared using Milli-Q IQ7000 model equipment with resistivity 18.2 MΩ·cm at 25 °C and total organic carbon (TOC) ≤ 5 ppb.
2. **7.5% DDM (w/v)**
  **Table:** 

Reagent	Final concentration	Amount
DDM powder	7.5%	0.75 g
Milli-Q H_2_O	n/a	Dissolved in 10 mL
  *Freeze at -20 °C.
3. **MelB_St_ dialysis buffer**
  **Table:** 

Reagent	Final concentration	Amount
NaCl (4 M in milli-Q H_2_O)	150 mM	37.5 mL
Tris-HCl (1 M, pH 7.5)	20 mM	20 mL
Glycerol (100%)	10% (v/v)	100 mL
DDM (10%) (w/v)	0.01% (w/v)	1 mL
Milli-Q H_2_O	n/a	To 1 L
4. **TBS (nanodiscs collection buffer)**
  **Table:** 

Reagent	Final concentration	Amount
NaCl (4 M in milli-Q H_2_O)	150 mM	37.5 mL
Tris-HCl (1 M, pH 7.5)	20 mM	20 mL
Milli-Q H_2_O	n/a	To 1 L
5. **TBS buffer with 5 mM imidazole (column binding buffer)**
  **Table:** 

Reagent	Final concentration	Amount
NaCl (4 M in milli-Q H_2_O)	150 mM	37.5 mL
Tris-HCl (1 M, pH 7.5)	20 mM	20 mL
Imidazole (1 M, pH 7.5)	5 mM	5 mL
Milli Q H_2_O	n/a	To 1 L
6. **TBS buffer with 25 mM imidazole (column washing buffer)**
  **Table:** 

Reagent	Final concentration	Amount
NaCl (4 M in milli-Q H_2_O)	150 mM	37.5 mL
Tris-HCl (1 M, pH 7.5) Imidazole (1 M, pH 7.5)	20 mM 25 mM	20 mL 25 mL
Milli-Q H_2_O	n/a	To 1 L
7. **TBS buffer with 300 mM imidazole (column eluting buffer)**
  **Table:** 

Reagent	Final concentration	Amount
NaCl (4 M in milli-Q H_2_O)	150 mM	37.5 mL
Tris-HCl (1 M, pH 7.5) Imidazole (1 M, pH 7.5)	20 mM 300 mM	20 mL 300 mL
Milli-Q H_2_O	n/a	To 1 L
*Note: All solutions are stored at 4 °C.*
8. **1 M Tris-HCl**
  1 M Tris base in milli-Q H_2_O was adjusted to pH 7.5, using concentrated HCl.
  **Table:** 

Reagent	Final concentration	Amount
Tris base	1 M	121.1 g
Milli-Q H_2_O		Dissolve to 900 mL
Concentrated HCl Milli-Q H_2_O		Adjust to pH 7.5
9. **1 M Imidazole**
  1 M Imidazole in milli-Q H_2_O was adjusted to pH 7.5, using concentrated HCl.
  **Table:** 

Reagent	Final concentration	Amount
Imidazole	1 M	68.08 g
Milli-Q H_2_O		Dissolve to 900 mL
Concentrated HCl Milli-Q H_2_O		Adjust to pH 7.5 and fill to 1 L

**Laboratory supplies**

1. 20 mL glass vials (DWK Life Sciences, catalog number: W224589)
2. Plastic conical tubes, 50 mL (Thermo Scientific, catalog number: 339653)
3. Nunc^TM^ serological pipettes, 5 mL (Thermo Scientific, catalog number: 170355)
4. 1.5 mL Eppendorf tubes (FisherBrand, catalog number: 05-408-129)
5. 2 mL Eppendorf tubes (FisherBrand, catalog number: 05-408-138)
6. Stir bars (FisherBrand, catalog number:14-513-82)
7. Transfer pipettes (VWR, catalog number: 16001-194)
8. Slider-A-Lyzer dialysis cassettes, 10 k MWCO (Thermo Scientific, catalog number: 66810)
9. Centrifugal concentrator Vivaspin 2, 50,000 MWCO PES (Sartorius, catalog number: VS0231)
10. Centrifugal concentrator Vivaspin 20, 5,000 MWCO PES (Sartorius, catalog number: VS2012)
11. INDIGO Ni-agarose resin, 50% slurry (Cube Biotech, catalog number: 75105)
12. TALON^®^ metal affinity resin (Takara, catalog number: 635503)
13. Poly-Prep chromatography column (Bio-Rad, catalog number: 731-1550)
14. Econo-Column^®^ chromatography column, 2.5 × 10 cm (Bio-Rad, catalog number: 737-2512)
15. Econo-Column funnel, 250 mL reservoir (Bio-Rad, catalog number: 731-0003)
16. SDS-12% gel in 1.5 mm thickness (prepared according to the instruction manual of Mini-PROTEAN Tetra Vertical Electrophoresis kit)

## Equipment

1. Branson ultrasonic cleaner (Branson, model: 2510)
2. Glass dewar flask for liquid nitrogen (FisherBrand, catalog number: 10-196-7)
3. Magnetic stirrer (Fisher Scientific, model: Isotemp)
4. Ultracentrifuge (Beckman Coulter, model: L-100XP Ultracentrifuge)
5. Eppendorf benchtop centrifuge (Eppendorf, model: 5417R)
6. Vortex mixer (Fisher Scientific, model: Analog Vortex Mixer, catalog number: 02215365)
7. Pipette controller (Drummond^TM^, model: Portable Pipet-Aid^TM^ XP)
8. Mini-PROTEAN Tetra Vertical Electrophoresis kit (Bio-Rad, catalog number: 165-8027FC)
9. NGC Quest FPLC system (Bio-Rad, model: NGC Quest 10 Plus, catalog number: 7880003)
10. Superdex^TM^ 200 Increase small-scale S columns, 10 × 300 mm (Cytiva, catalog number: 28990944)
11. Spectrophotometer (Thermo Fisher, model: Genesys 10-S UV-Vis)

## Procedure

**Figure 4.**
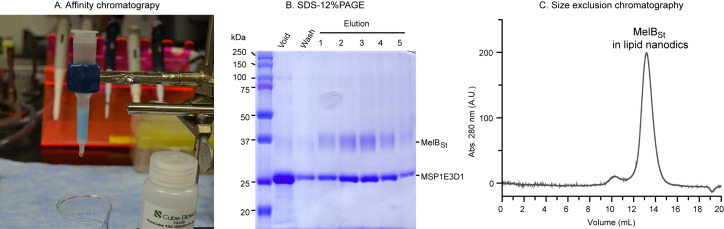
Separation of the mixed nanodiscs sample by Ni-affinity chromatography. A. Illustration of the gravity flow chromatography using a column packed with INDIGO Ni-agarose resin. B. SDS-12%PAGE analysis. From each fraction, 10 μL were loaded and the gel was stained by Coomassie blue. C. Size exclusion chromatography shows the major peak of the reconstituted MelB_St_ in lipid nanodiscs is sharp and symmetric.

- **Prepare 5 mL fresh lipid mixture at 40 mg/mL in 7.5% DDM solution**
  1. Weigh 200 mg of *E. coli* polar lipids extract powder using a pre-weighed 50 mL plastic conical tube.
  2. Add 4.5 mL of 7.5% DDM (see Recipe 2) to the dry lipid.
  3. Vortex the mixture for 2–3 min.
**Sonicator setup**: Fill the sonicator with ice-cold 0.001% Triton X100 bath liquid (see Recipe 1) and adjust the water level to achieve the maximum strength of sonication.
  4. Place the tube into the sonicator tank and sonicate the suspension for 1 min. During sonicating, pipette the suspension up and down using a 5 mL plastic serological pipette on a portable pipette controller. Use the same pipette until the end of step A7.
  5. Cool on ice for 5 min.
  6. Repeat steps A4–5 until completely dissolved. It might take 5–7 cycles. An optional 1 min vortex can also be applied at the beginning of each cycle.
*Note: Avoid overheating. Shipping freezer packs can be used during intervals to cool the bath liquid. Maintain ice-cold bath liquid to the optimal level and top it off if required.*
  7. Measure the volume of the suspension using the same 5 mL pipette and adjust the volume to 5 mL using 7.5% DDM solution to obtain 40 mg/mL.
  8. Conduct the freeze-thaw-sonicate cycle 3–4 times until the lipid mixture is translucent.
    - Freeze the 5 mL lipid samples in the same tube by plunging and rotating the tube into a glass dewar flask containing liquid nitrogen; hold the tube at an angle to provide more surface area to create a thin layer of frozen lipids around the wall of the tube. The purpose of the thin layer is not only to make thawing faster in the next step but also to make sure the entire suspension is completely frozen (Figure 2A).
    - Thaw at room temperature until the lipid sample becomes liquid. Typically, this may take approximately 5–10 min with shaking; allow a brief warming period (~5 s) by hand.
    - Sonicate the liquid sample for 30 s.
    - Cool on ice for 5 min before the next freeze-thaw-sonicate cycle.
  9. Keep the translucent lipid mixture at room temperature for the next step (Figure 2B).
  10. Aliquot the excess lipid mixture by 0.5 mL into 1.5 mL Eppendorf tubes, flash freeze in liquid nitrogen, and store at -80 °C.
    **Figure 2.**
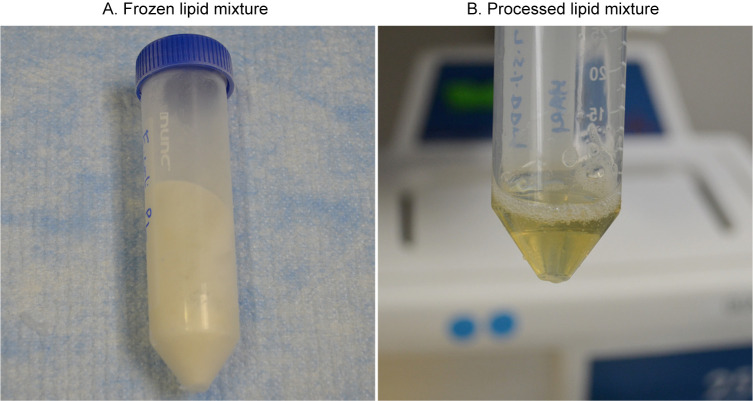
Lipids preparation. A. Frozen detergent-stabilized lipid suspension on the wall of the 50 mL tube. B. Processed pale-yellowish translucent lipid suspension after freeze-thaw-sonicate cycles.
*Note: When using the frozen lipids from -80 °C, thaw them at room temperature, sonicate for 30 s, and leave the sample at room temperature for immediate use. The lipids can be frozen again with liquid nitrogen and used 2–3 times.*
- **Bio-Beads preparation**
*Note: Prepare the Bio-Beads before setting up the nanodisc reconstitution.*
  1. Pack the ~20–25 g of Bio-Beads in a Bio-Rad glass Econo-Column^®^ cartography column (2.5 × 10 cm, volume ~50 mL) fitted with a 250 mL solution reservoir funnel.
  2. Wash once with 10 volumes (~200–250 mL) of methanol and let it drain completely.
  3. Wash once with 10 volumes (~200–250 mL) of ethanol and let it drain completely.
  4. Wash 10 times with 10 volumes (2–2.5 L) of milli-Q water.
  5. Remove the funnel and transfer the prepared Bio-Beads in milli-Q water into a 50 mL plastic conical tube and store at 4 °C. The prepared Bio-Beads samples in milli-Q water can be stored for 4–6 months.
- **Reconstitution of nanodiscs**
  Required materials:
  - 40 mg/mL *E. coli* lipids in 7.5% DDM (see Recipe 2).
  - MelB_St_ dialysis buffer (see Recipe 3).
  - Purified His-tagged MelB_St_ in the defined MelB dialysis buffer.
  - Purified and TEV-treated membrane scaffold protein MSP1E3D1∆His (alternate source: Cube Biotech, catalog number: 26162).
  - 20 mL glass vial with cap.
  1. Mixing. Prepare a suspension containing 6.65 mM *E. coli* lipids and 1 mg/mL His-tagged MelB_St_, corresponding to a ratio of His-tagged MelB_St_ to lipids of 1:350 mole/mole.
    - In a 20 mL glass vial, take 1 mL of His-tagged MelB_St_ at 1 mg/mL concentration (~19 μM).
    - Add 133 μL of 50 mM lipids and mix using pipettes with no vortex.
    - Incubate the mixture for 10 min at room temperature.
    - Add 365 μL of 260 μM MSP1E3D1∆His to the MelB_St_/lipid mixture, bringing the final concentration to 95 μM and a ratio of MSP1E3D1∆His to MelB_St_ of 5:1.
    - Incubate the mixture (Figure 3A) at room temperature with intermittent mixing for 30 min.
*Note: The mixture should be clear at the end of the 30 min incubation, indicating no protein precipitation (see General note 1 and 2).*
    **Figure 3.**
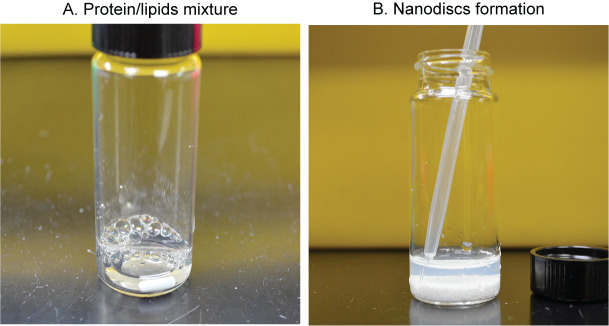
Reconstitution procedure of MelB_St_ nanodiscs. A. Mixing the purified MelB_St_ and MSP1E3D1∆His protein with the detergent-destabilized phospholipids at 1:5:350 ratios, respectively. B. Collecting the slightly turbid suspension containing MelB_St_-reconstituted lipid nanodiscs and the empty nanodisc without MelB_St_.
  2. Detergent removal by incubation with Bio-Beads (1 mg of MelB_St_ may need ~ 800 mg beads).
    - Weigh the prepared Bio-Beads using a pre-weighed 2 mL microfuge tube after removing the water using a transfer pipette.
    - Wash the Bio-Beads with 1 mL of TBS buffer (see Recipe 4) by mixing and removing the buffer three times.
    - Add 2/3 of treated Bio-Beads (~500 mg) to the lipids/protein mixture (Figure 3B).
    - Incubate at 4 °C for 2 h with mild stirring.
    - Add the remaining Bio-Beads (~300 mg).
    - Continue the incubation overnight at 4 °C under mild stirring/mixing.
*Note: The nanodiscs should be formed after removing the detergents with the Bio-Beads, and the suspension should be cloudy, indicative of nanodiscs (Figure 3B).*
    - Transfer the nanodiscs-containing slightly turbid suspension into a fresh 1.5 mL Eppendorf tube using a transfer pipette (or gel-loading tips) (Figure 3B).
    - Centrifuge at 20,000× *g* for 10 min at 4 °C to remove the traces of contaminating Bio-Beads.
    - Transfer the nanodiscs-containing supernatant into a fresh 2 mL microfuge tube.
*Note: At this step, the nanodiscs may contain one or two molecules of MelB_St_ per disc, or they may be empty discs with no MelB_St_. The nanodisc samples are in the TBS buffer after completely removing the detergents.*
*Note: In the cryo-EM single-particle analysis, we observed the majority of loaded nanodisc containing one molecule of MelB_St_. Although its heterogeneity interferes with determining protein concentrations, SDS-PAGE analysis can analyze the protein species and their stoichiometry.*
  3. Isolation of MelB_St_-reconstituted nanodiscs by affinity chromatography.
*Note: The empty nanodiscs with no His-tag and the His-tagged MelB_St_ nanodiscs can be separated by affinity chromatography using Indigo Ni-agarose resin (Figure 4A). The empty nanodiscs are in the void, and the His-tagged MelB_St_ nanodiscs are adsorbed by the Ni-NTA beads. We usually conduct this separation using a gravity column at room temperature. However, the following steps can be performed at 4 °C and/or using pre-packed affinity chromatography columns on an FPLC system.*
    - Pack 2 mL of 50% INDIGO Ni-agarose resin to a 1 mL bed volume on a chromatography column.
    - Wash the column using 10 mL (10× volume) of milli-Q water.
    - Wash the column using 10 mL of the TBS buffer containing 5 mM imidazole (see Recipe 5).
    - Add 1 M of imidazole solution into the nanodisc mixture to a final concentration of ~5 mM.
    - Load the nanodiscs mixture onto the washed column.
    - Wash the column with 10 mL (10× volume) of 5 mM-imidazole TBS buffer (see Recipe 5).
    - Wash the column twice with 10 mL of 25-mM imidazole TBS buffer (see Recipe 6) to remove the empty nanodiscs without a His tag.
    - Elute the bound His-tagged MelB_St_ nanodiscs with 10 mL of 300-mM imidazole TBS buffer (see Recipe 7).
    - Immediately collect fractions at each 0.5 mL in 1.5 mL Eppendorf tubes.
    - Pool the protein-containing fractions (judged by 280 nm absorption using the elution buffer to blank).
    - Transfer all pooled solution into a 2 mL Vivaspin 2 centrifugal concentrator 50,000 MWCO PES and centrifuge at 3,000× *g* for 5 min at 4 °C. Mix the solution using a transfer pipette before starting another centrifugation at the same condition until the volume reduces to ~0.5 mL to obtain the protein concentration to ~3 mg/mL.
    - Dialyze MelB_St_ nanodiscs against the TBS buffer (see Recipe 4) overnight using a dialysis cassette with 10,000 MWCO.
*Note: Estimation of protein concentration: using a spectrophotometer, measure A_280_ nm absorbance value, which is used in the following equation:*
    Protein concentration, C (M) = A_280 nm_/extinction coefficient () × light path-length (*l*),
    = 135110 (https://web.expasy.org/protparam/) based on 1 MelB_St_: 2 MSP1E3D1∆His per nanodisc.
*l* = 1 when using a 1 cm cuvette

## Data analysis

1. SDS-12% PAGE. An aliquot of protein fraction (10 μL) from the Ni-affinity chromatography was analyzed using SDS-12% PAGE and stained with Coomassie blue (Figure 4B). The 54 kDa His-tagged MelB_St_ migrated to 37 kDa, and the 30 kDa His-tag-removed MSP1E3D1 migrated slightly above 25 kDa. The unbound fraction in the void shows a strong MSP1E3D1 band and weak MelB_St_ band, which indicates that most nanodiscs have no reconstituted MelB_St_. The elution fractions indicate the great enrichment of MelB_St_-containing nanodisc with a stochiometric ratio of 1:2 with MSP1E3D1.
2. Size exclusion chromatography. Load 0.5 mL of concentrated and dialyzed nanodiscs onto a Superdex 200 Increase (10 × 300 mm) column pre-equilibrated with TBS (see Recipe 4) on a Bio-Rad NGC Quest FPLC system. Figure 4C presents the chromatogram of the MelB_St_ nanodiscs. The chromatogram peak fractions collected using the BioFrac fraction collector can be directly used for downstream applications such as biochemical studies [15,18], cryo-EM single-particle analysis [17], etc., as described in the validation section.

## Validation of protocol

**Figure 5.**
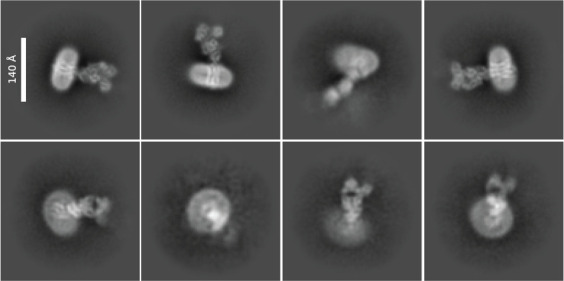
2D class averages. Representative data from the study was described in the *eLife* article [17]. The sample containing the wild-type MelB_St_ in lipid nanodiscs using MSP1E3D1∆His, the MelB_St_-specific Nb725_4, NabFab, and anti-Fab Nb were used for imaging using Titan Krios TEM with a K3 detector of S^2^C^2^, Stanford, CA, as described [17]. The image processing, particle pick, and 2D classification were performed in CryoSPARC program. Upper row: side view of the nanodiscs carrying the MelB_St_ complex; each class contains 4,000–7,000 particles. Lower row: top view of nanodiscs carrying MelB_St_ complex; each class contains 1,000–4,000 particles. Bar size, 140 Å.

This protocol or parts of it have been used and validated in the following research article(s):

In the *J Gen Physiol* article [18], MelB_St_ nanodiscs were prepared to test the binding of MelB regulatory protein. In the *J Mol Biol* article [15], a few single-site mutations of MelB_St_ largely decreased protein stability, preventing the downstream biophysical analysis. Reconstitution into lipid nanodiscs allowed cation binding and substrate binding assays to be possible. Figure 5 shows different orientations of nanodiscs carrying MelB_St_ in complex with the nanobody725_4, NabFab, and anti-Fab Nb. In the *eLife* article [17], MelB_St_ was reconstituted into nanodiscs, which increased protein stability and single-particle picking.

## General notes and troubleshooting

**General notes**

Tips for reconstituting other membrane proteins:

1. An essential modification in Section C is to use a protein-specific buffer to maintain membrane protein stability at the mixing step. The most important component is to use the detergent optimal to the membrane protein of interest. The detailed detergent study in [24] contains useful information for guiding detergent selection.
2. In the mixing step of Section C, the concentration of the membrane proteins should be < 1 mg/mL.

**Troubleshooting**

Problem 1: Protein precipitation during the mixing step.

Possible cause: The quality of the protein sample is poor.

Solution: A) Perform all incubations on the ice. B) Optimize the membrane protein buffer and dilute the protein if necessary. C) Increase the ratio of MSPs with the membrane protein up to 10:1. D) Select a suitable MSP type according to the size and hydrodynamic radius of the membrane protein.

Problem 2: No membrane protein with nanodiscs.

Possible cause: Protein aggregations or incompatibility with the selected lipids mixture.

Solution: A) The protein may require a different lipid composition. Lipid selection is important. The lipid components of the prokaryotic and eukaryotic membranes are different. The *E. coli* polar lipids extract (Avanti, catalog number: 100500P) can be first selected to test for most bacterial membrane proteins. More information on lipids selection is available in [25]. However, a trial-and-error approach is often needed to identify optimal lipid composition.

**Laboratory safety**

1. Use proper personal protective equipment (PPE) and adhere to general cryogenic safety procedures while handling liquid nitrogen. Transfer liquid nitrogen in well-ventilated areas to prevent generating low-oxygen surroundings and causing breathing health hazards.
2. Review the MSDS provided by the manufacturer for all chemicals and materials.
